# Commentary: Yawning, acute stressors, and arousal reduction in Nazca booby adults and nestlings

**DOI:** 10.3389/fpsyg.2015.01654

**Published:** 2015-10-29

**Authors:** Andrew C. Gallup, Anne B. Clark

**Affiliations:** ^1^Department of Psychology, State University of New York at OneontaOneonta, NY, USA; ^2^Department of Biological Sciences, Binghamton UniversityBinghamton, NY, USA

**Keywords:** yawning, stress, corticosterone, contagious yawning, thermoregulation

Liang et al. (2015) recently reported on the relationship between yawning and stress responses in a wild population of Nazca boobies (*Sula granti*) in the Galapagos. Their analysis covered two separate investigations: a human capture-restraint stressor applied to adult boobies, and observations of nestlings maltreated by Non-parental Adult Visitors (NAVs). The authors conclude that the temporal sequence of yawning following these stressors provides support for the newly termed arousal reduction hypothesis, an idea initially proposed by Dourish and Cooper (1990), and demonstrates a communicative function to yawning. This study adds much-needed field data on patterns of yawning, stress and corticosterone in a natural context. Our commentary addresses the inference of a communicative function of yawning, discusses the match between the reported patterns and a thermoregulatory function, and draws attention to important shortcomings of the arousal reduction hypothesis as an explanation of yawning.

The authors state in the abstract that they “tested the hypothesis that yawning communicates to others a transition from a state of physiological and/or psychological arousal (for example, due to action of a stressor) to a more relaxed state” (p. 38), i.e., the arousal reduction hypothesis. This is quite misleading as the experiments reported were not designed as a direct test of this hypothesis, which is proposed later in the discussion. Useful development of the hypothesis would include a sketch of how it would work in the context of the results. In fact, the reported patterns—nocturnal yawning by adult boobies and particularly yawning by nestlings in the absence of parents or responses by nearby nestlings—raise doubts about evolved signal function, which generally requires selection in terms of receivers and senders (e.g., Bradbury and Vehrencamp, 2011).

Liang et al. (2015) state that their results on nestlings support the arousal reduction hypothesis and challenge the thermoregulatory hypothesis that yawning is a cooling mechanism for the brain (Gallup and Gallup, 2007). Despite critiques (e.g., Guggisberg et al., 2010), the primary predictions of the thermoregulatory hypothesis have been supported in different model systems (see recent review and discussion of critiques: Gallup and Eldakar, 2013) and we think it remains an important possibility here. While admitting that their data on adults cannot speak to the role of thermoregulation, the authors reject it based on the positive correlation between the nestlings' latency to stand and latency to yawn following attacks by NAVs and the argument that this relation is inconsistent with the thermoregulatory hypothesis. They assume that nestlings waiting longer to stand are equally hyperthermic to those that stand right away; these nestlings just endure hyperthermia for a greater length of time. The hyperthermic stress response of Nazca booby nestlings is, however, likely to be individually variable, as reported in a laboratory study of handling stress and yawning in another bird, the budgerigar (*Melopsittacus undulatus*) (Miller et al., 2010). Wide individual variation in budgerigars' under-wing temperatures during handling restraint correlated with variation in post-stress timing of yawning, with the more hyperthermic budgerigars yawning sooner after stress. It would be consistent if the more heat-stressed booby nestlings both stood earlier after NAVs left and yawned sooner to cool down. Data on variation in hyperthermic responses of Nazca booby nestlings are critical for disentangling these alternative hypotheses, which assume similar neuroendocrine responses to stress, but differ in terms of the mechanisms controlling yawning (neuroendocrine factors vs. elevated temperature) and the function yawns serve (communication vs. brain-cooling). Individual profiles of temperature and perhaps cortisol at the end of an attack and at the first yawn might discriminate the triggers. Note that the correlated occurrence of yawning and other thermoregulatory responses (i.e., standing, gular fluttering) post-stressor is consistent with a thermoregulatory explanation. Budgerigars also showed a positive correlation of yawning with wing-venting and gular fluttering under thermal stress (Gallup et al., 2010).

Liang et al.'s (2015) own explanatory hypothesis, the arousal reduction hypothesis, brings its own logical challenges. It is based on the observation that, following a threat, booby yawns increased in frequency only after a time delay, and after corticosterone levels in adults subsided. Expanding upon earlier ideas presented by Dourish and Cooper (1990), the authors further propose that yawns “signal” the down-regulation of arousal to others. Dourish and Cooper (1990) themselves did not push the communication function, apparently using the term “signal” in a clinical sense and only noting that social effects were known for humans. Just because yawning in boobies provides information about stress to human observers does not mean it serves as a social signal to conspecifics. A proper understanding of yawning must acknowledge that the behavior is subject to cost-benefit tradeoffs (e.g., it might bring on a new attack by NAVs and be suppressed until the falling risk is balanced by its benefits). Multiple evolved functions, e.g., thermoregulation and signaling, are possible, even likely, but these add to the animal's challenge of balancing costs and benefits, and our challenge to account for observable behavior.

Ideally, a new hypothesis illuminates and perhaps accounts for previously documented effects better than standing theories, but at the least, it should offer a better, internally coherent account of the data on which it is based. In this case, the arousal reduction hypothesis offers no clear explanation for previously reported empirical patterns, for example, yawning and ambient temperature variation (see Gallup, 2015) or high frequencies of yawning after waking (e.g., Provine et al., 1987). But it also offers an insufficient account of costs and benefits for yawning and signaling to explain the patterns reported in this paper.

Overall we believe the results reported by Liang et al. (2015) are important and interesting. As they stand, they cannot, however, support a communicative function to yawning and they appear to be consistent with the thermoregulatory hypothesis. The newly described arousal reduction hypothesis is at this point causally and functionally unclear; it fails to stipulate the benefits of yawning or identify who receives such benefits. It also fails to illuminate patterns of yawning documented in the literature. We hope this commentary spurs further analysis of existing data as well as future research investigating the functions of yawning.

## Conflict of interest statement

The authors declare that the research was conducted in the absence of any commercial or financial relationships that could be construed as a potential conflict of interest.
